# PI3K p110β isoform synergizes with JNK in the regulation of glioblastoma cell proliferation and migration through Akt and FAK inhibition

**DOI:** 10.1186/s13046-016-0356-5

**Published:** 2016-05-12

**Authors:** Hua-Fu Zhao, Jing Wang, Hao-Ran Jiang, Zhong-Ping Chen, Shing-Shun Tony To

**Affiliations:** Department of Health Technology and Informatics, The Hong Kong Polytechnic University, Hong Kong, China; Department of Neurosurgery/Neuro-oncology, Sun Yat-sen University Cancer Center; State Key Laboratory of Oncology in South China; Collaborative Innovation Center for Cancer Medicine, Guangzhou, 510060 China; Department of Neurosurgery and Shenzhen Key Laboratory of Neurosurgery, Shenzhen Second People’s Hospital, Shenzhen University 1st Affiliated Hospital, Shenzhen, 518035 China; Department of Neurosurgery, Huizhou First People’s Hospital, Huizhou, 516003 China

**Keywords:** Glioblastoma, PI3K, p110β, JNK, Synergism, Proliferation, Migration

## Abstract

**Background:**

Glioblastoma multiforme is the most aggressive malignant primary brain tumor, characterized by rapid growth and extensive infiltration to neighboring normal brain parenchyma. Both PI3K/Akt and JNK pathways are essential to glioblastoma cell survival, migration and invasion. Due to their hyperactivation in glioblastoma cells, PI3K and JNK are promising targets for glioblastoma treatment.

**Methods:**

To investigate the combination effects of class I_A_ PI3K catalytic isoforms (p110α, p110β and p110δ) and JNK inhibition on tumor cell growth and motility, glioblastoma cells and xenografts in nude mice were treated with isoform-selective PI3K inhibitors in combination with JNK inhibitor.

**Results:**

We showed that combined inhibition of these PI3K isoforms and JNK exerted divergent effects on the proliferation, migration and invasion of glioblastoma cells in vitro. Pharmacological inhibition of p110β or p110δ, but not p110α, displayed synergistic inhibitory effect with JNK inhibition on glioblastoma cell proliferation and migration through decreasing phosphorylation of Akt, FAK and zyxin, leading to blockade of lamellipodia and membrane ruffles formation. No synergistic effect on invasion was observed in all the combination treatment. In vivo, combination of p110β and JNK inhibitors significantly reduced xenograft tumor growth compared with single inhibitor alone.

**Conclusion:**

Concurrent inhibition of p110β and JNK exhibited synergistic effects on suppressing glioblastoma cell proliferation and migration in vitro and xenograft tumor growth in vivo. Our data suggest that combined inhibition of PI3K p110β isoform and JNK may serve as a potent and promising therapeutic approach for glioblastoma multiforme.

**Electronic supplementary material:**

The online version of this article (doi:10.1186/s13046-016-0356-5) contains supplementary material, which is available to authorized users.

## Background

Glioblastoma multiforme (GBM) is the most common aggressive and malignant primary tumor in the central nervous system. GBM is classified as WHO grade IV glioma, characterized by rapid growth and extensive infiltration to neighboring normal brain parenchyma, which prevents complete surgical resection and leads to tumor recurrence and poor prognosis [1, 2]. Thus, migration and invasion of glioblastoma cells are potential therapeutic targets for GBM treatment.

Phosphatidylinositol 3-kinases (PI3Ks) are lipid kinases involved in a variety of biological responses including cell proliferation metabolism, motility, survival and angiogenesis [3]. Inhibition of PI3K activity suppresses formation of lamellipodia and membrane ruffles, indicating PI3K/Akt pathway may regulate cell migration through mediating actin filament remodeling [4–6]. Class I_A_ PI3K is activated by receptor tyrosine kinases (RTKs) and is composed of a heterodimer consisting of a 110 kDa catalytic subunit (p110α, p110β and p110δ) and a 85 kDa regulatory subunit [3]. The pan-PI3K inhibitors wortmannin and LY294002 are limited in clinical use due to their poor pharmaceutical properties, unacceptable toxicities and off-target effects. Although a number of novel pan-PI3K inhibitors like BKM120, PX866 and XL147 are currently in clinical trials, selective inhibitors for p110 isoforms that may display less off-target effects and toxicities are attractive options for GBM treatment [7]. Recently, The p110α-selective inhibitor BYL719 and the p110δ-selective inhibitor CAL-101 have already entered phase I/II clinical trials and exhibited moderate anti-proliferative and pro-apoptotic activities in solid tumors or hematologic malignancies [8, 9]. Therefore, PI3K p110 isoforms are promising targets for GBM treatment.

Considering that inhibition of PI3K isoforms might be compensated by other signaling pathways which subsequently compromises the inhibitory effects, it is critical to investigate combination therapy through targeting PI3K and other molecules. The c-Jun N-terminal kinase (JNK) plays important roles in the apoptosis and motility of cancer cells, and has crosstalk with Akt signaling. A number of studies find that JNK activation is frequently accompanied by activation of Akt and PI3K in tumor cells upon *PTEN* loss or epidermal growth factor receptor (EGFR) overexpression [10–12]. In addition, JNK can be activated by growth factors and G protein–coupled receptors (GPCRs) and is constitutively activated in glioblastoma, indicating that the JNK pathway may have crosstalk with PI3K/Akt pathway, and they may share the same upstream signaling components [13, 14]. Therefore, combined inhibition of class I_A_ PI3K catalytic isoforms and JNK may have synergistic effect on glioblastoma cells.

Here we demonstrated that isoform-selective PI3K inhibitors and JNK inhibitor exhibited divergent effects on the proliferation, migration and invasion of glioblastoma cells in vitro. Inhibition of p110β or p110δ, but not p110α, exerted synergism with JNK on impeding glioblastoma cell proliferation and migration through decreasing Akt, focal adhesion kinase (FAK) and zyxin phosphorylation, resulting in the blockade of lamellipodia and membrane ruffles formation. Further, combined inhibition of p110β and JNK effectively reduced xenograft tumor growth in vivo. These results suggested that combined inhibition of p110β and JNK may be an effective therapy for glioblastoma treatment.

## Methods

All experimental protocols used in this study were approved by the Hong Kong Polytechnic University Health and Safety Committee and the Ethics Review Board of Sun Yat-sen University Cancer Center.

### Cell culture

Normal human astrocytes cell line was purchased from ScienCell Research Laboratories. Human glioblastoma cell lines U87-MG and U-373 MG were obtained from ATCC. Cells were cultured in Minimum Essential Medium Alpha (α-MEM) (Gibco) supplemented with 10 % (v/v) fetal bovine serum (FBS) (Gibco). Cells were incubated at 37 °C in 5 % CO_2_ atm.

### Reagents and antibodies

Monoclonal anti-Akt (#9272) anti-phospho-Akt (Ser473) (#9271), anti-phospho-Akt (Thr308) (#9275), anti-SAPK/JNK (#9258), anti-phospho-SAPK/JNK (Thr183/Tyr185) (#9251), anti-c-Jun (#9165), anti-phospho-c-Jun (Ser63) (#2361), anti-FAK (#3285), anti-phospho-FAK (Tyr925) (#3284), anti-zyxin (#3553), anti-phospho-zyxin (Ser142/143) (#8467), anti-GAPDH (#2118) and horseradish peroxidise (HRP)-conjugated secondary antibodies were purchased from Cell Signaling Technology. Polyclonal anti-β-actin (sc-1616) were obtained from Santa Cruz Biotechnology. CAL-101, PIK-75 and TGX-221 were obtained from Selleck Chemicals. SP600125 was from Sigma-Aldrich. Drug treatment was generally performed in α-MEM medium supplemented with 10 % FBS, unless the additional illustration.

### Cell proliferation assay

Cells were seeded onto 96-well plates (2000 cells per well). On the next day cells were treated with inhibitors for 48 h. The 3-(4,5-Dimethylthiazol-2-yl)-2,5-diphenyltetrazolium bromide (MTT) assay was performed by adding 20 μL of MTT to each well followed by incubation for 4 h at 37 °C. The formazan crystal was subsequently dissolved in 150 μL of dimethyl sulfoxide (DMSO). Absorbance at 570 nm was determined by Benchmark Plus™ microplate spectrophotometer (BIO-RAD). Combination effect was evaluated by combination index (CI) as described by Chou [15]. Fraction affected (FA) refers to the inhibition of cell proliferation and is calculated by: FA = 1- (% cell proliferation/100). According to the FA values, CI was calculated by Compusyn software. CI <0.9 indicates synergistic effect; CI >1.1 indicates antagonistic effect; CI between 0.9 and 1.1 indicates additive effect. Experiments were carried out for at least three times and each independent experiment consisted of four replicates.

### Wound healing assay

Glioblastoma cells were seeded onto 12-well plates (3 × 10^5^ cells per well) and then incubated for 24 h to achieve 90-100 % confluence. Cells were pretreated with 5 μg/mL mitomycin C for 1 h to eliminate the interference by cell proliferation. Wounds on the confluent cells were created using a sterile 200 μL pipette tip. After rinsed with phosphate-buffered saline (PBS) for 3 times cells were treated with inhibitor in α-MEM medium supplemented with 5 % FBS and then incubated for 24 h at 37 °C. Cells were photographed immediately after inhibitor treatment (time zero) and at 24 h after wounding. Cell migration rate was indicated as the number of cells migrating into the original wounds.

### Immunofluoresescence

Glioblastoma cells were seeded onto sterile coverslips in 24-well plates (2.5 × 10^4^ cells per well) and incubated for 24 h at 37 °C. After 3-h inhibitor treatment cells were fixed with 4 % paraformaldehyde for 15 min and then permeabilized with 0.25 % Triton X-100 for 10 min at room temperature. Coverslips were blocked with 1 % bovine serum albumin (BSA) for 30 min, then incubated with Alexa Fluor 594-conjugated phalloidin (Invitrogen) for 20 min to visualize actin filaments. Cells were air dried and mounted with ProLong Gold antifade mountant with DAPI stain (Invitrogen). Lamellipodia were analyzed under Leica TSC SP8 confocal laser scanning microscope and membrane ruffles were observed by phase contrast microscopy. In each case, about 200 cells were photographed and representative cells were shown. Independent experiments were carried out in triplicate.

### Invasion assay

Invasiveness of glioblastoma cells was determined using BioCoat™ Matrigel™ Invasion Chamber (BD Bioscience). Briefly the transwell inserts with 8 μm pores and pre-coated Matrigel was rehydrated for 2 h at 37 °C. U-87 MG cells were pretreated with inhibitor in serum-free medium for 1 h at 37 °C prior to seeding onto the insert (2.5 × 10^4^ cells per insert). Medium supplemented with 5 % FBS in each well of companion plate was served as a chemoattractant. After 24-h incubation, the matrigel layer and non-invasive cells in upper surface of the membrane was removed. Cells in the lower surfaces of the membrane were fixed with absolute methanol and were then stained with 0.1 % crystal violet solution. Cells were photographed under a light microscope at 100× magnification and invasive cells from at least 10 representative fields were counted using ImageJ software. Independent experiments were carried out in triplicate.

### Western blotting

Total proteins were extracted by RIPA lysis buffer and protein concentrations were determined using the BCA protein assay (Thermo Scientific). Proteins were then separated by 8 % SDS-PAGE and transferred to PVDF membranes (Millipore). After blocking with 5 % non-fat milk or 5 % BSA for 2 h membranes were incubated with gentle agitation in primary antibodies (1:1000) overnight at 4 °C and then in HRP-conjugated secondary antibodies (1:5000) for 1 h at room temperature. Positive signals were visualized by ECL chemiluminescence using ChemiDoc MP Imaging System (Bio-Rad).

### Tumor xenograft for combination treatment in vivo

For in vivo tumor growth study 100 μL of U-87 MG cells (5 × 10^6^ cells) were subcutaneously injected into the lower flanks of 5-week-old Balb/C nude mice (female, 16–20 g, Vital River Laboratories). Once the tumors reached an average volume of ~70-80 mm^3^, mice (*n* = 6 of each group) were intraperitoneally injected once daily for 7 days with vehicle, TGX-221 (40 mg/kg) and CAL-101 (20 mg/kg) alone, or in combination with SP600125 (40 mg/kg). Ten percent DMSO (v/v), 30 % polyethylene glycol 400 (v/v), 1 % Tween 80 (v/v) and 59 % saline (v/v) was used as vehicle control. Tumor volumes were determined by caliper measurement every 2–3 days, and calculated by the formula: Volume = (width)^2^ x length/2, where length is the longest diameter and width is the shortest diameter perpendicular to length.

### Statistical analysis

Data were presented as mean ± S.E.M and analyzed by SPSS v22.0. Curves and histograms were drawn using GraphPad PRISM v5.0. Statistical comparisons among groups in proliferation invasion and migration assays were evaluated by One-way ANOVA and Post Hoc multiple comparison Tukey HSD test. For in vivo study, comparisons of tumor volumes among groups were assessed by One-way repeated measures ANOVA and Post Hoc multiple comparison Tukey HSD test. The difference was considered to be significant at *p* <0.05.

## Results

### Isoform-selective PI3K inhibitors suppress glioblastoma cell proliferation and Akt signaling

**Fig. 1:**
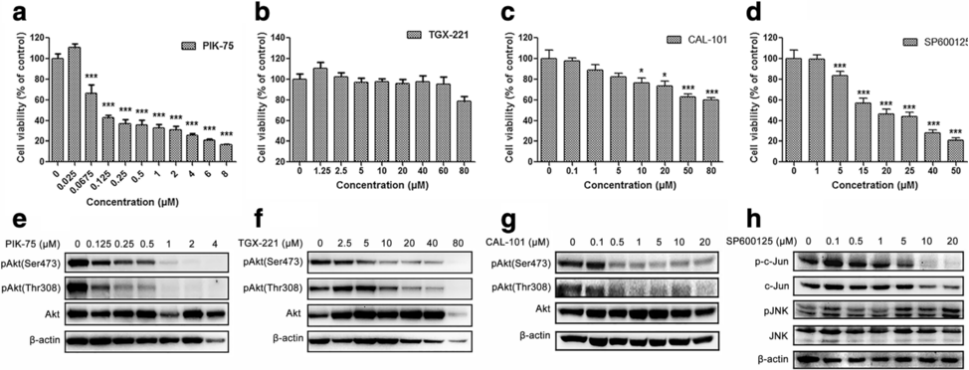
Inhibitory effects of PIK-75, TGX-221, CAL-101 and SP600125 on glioblastoma cell proliferation and signaling transduction. **a**-**d** U-87 MG cells were treated with PIK-75, TGX-221, CAL-101 or SP600125 at different concentrations for 48 h. DMSO was used as a carrier control. Cell viability of U-87 MG cells sharply decreased when treated with PIK-75, CAL-101 and SP600125, whereas TGX-221 had no significant inhibitory effect (*n* = 3; *p* values were determined by One-way ANOVA and Post Hoc multiple comparison Tukey HSD test. *: *p* <0.05; **: *p* <0.01; ***: *p* <0.001). **e**-**g** Isoform-selective PI3K inhibitors PIK-75, TGX-221 and CAL-101 dramatically inhibited Akt phosphorylation at Ser473 and Thr308. **h** JNK inhibitor SP600125 suppressed JNK signaling via reducing c-Jun phosphorylation, whereas JNK phosphorylation was not affected

Three PI3K inhibitors PIK-75 TGX-221 and CAL-110, with selectivity towards p110 catalytic isoforms p110α, p110β and p110δ respectively, were employed in this study (Additional file 1: Table S1). We first investigated the effects of these inhibitors on cell proliferation in glioblastoma cell line U-87 MG. Cells were incubated with PIK-75, TGX-221 and CAL-101 respectively at various concentrations for 48 h. The p110α inhibitor PIK-75 displayed dose-dependent inhibitory effect on U-87 MG cell proliferation and Akt signaling (Fig. 1a, e). Cell viability of U-87MG cells was reduced significantly upon PIK-75 treatment (≥0.0675 μM) (Fig. 1a). In contrast, although the phosphorylation levels of Akt at both serine 473 (Ser473) and threonine 308 (Thr308) residues were reduced after the treatment, the p110β inhibitor TGX-221 did not show any significant inhibitory effect, indicating that p110β possesses kinase-independent function to maintain cell viability (Fig. 1b, f). Compared with PIK-75, the p110δ inhibitor CAL-101 also suppressed U-87 MG cell proliferation and Akt phosphorylation in a dose-dependent manner but was less effective (Fig. 1c, g). The values of half maximal inhibitory concentration (IC_50_) of PIK-75, TGX-221 and CAL-101 were 0.09 μM, 484.82 μM and 39.11 μM respectively (Additional file 1: Figure S1). The inhibitory effects of these three inhibitors on another glioblastoma cell line U-373 MG were similar to U-87 MG cells (Additional file 1: Figure S2).

### Combined inhibition of p110β/δ and JNK exerts synergistic effect on blockade of glioblastoma cell proliferation in vitro

SP600125 is a JNK inhibitor with broad spectrum for JNK1 JNK2 and JNK3 (Additional file 1: Table S2). We analyzed the effect of SP600125 on U-87 MG cell proliferation and JNK signaling. Similar to PIK-75 and CAL-101, SP600125 significantly suppressed cell proliferation in a dose-dependent manner with an IC_50_ of 20.43 μM (Fig. 1d, Additional file 1: Figure S1). SP600125 decreased phosphorylation level of c-Jun, a substrate of JNK, whereas JNK phosphorylation was not affected (Fig. 1h).

**Fig. 2:**
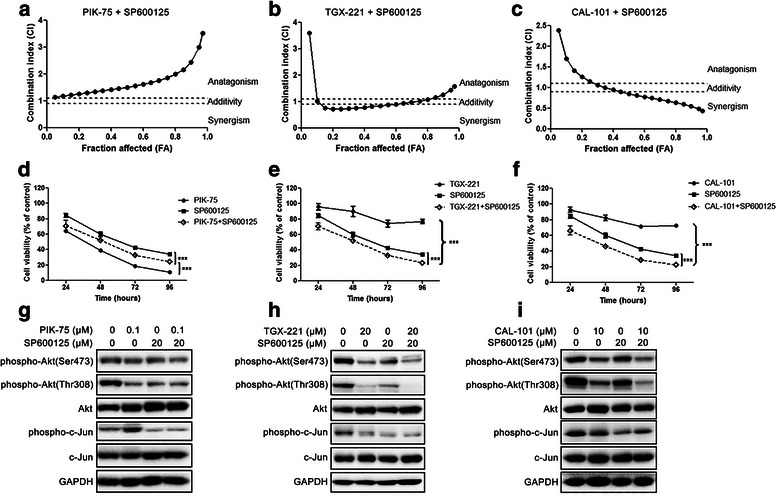
Combination effects of isoform-selective PI3K inhibitors and SP600125 on glioblastoma cell proliferation. **a**-**c** FA-CI plots were generated using Chou-Talalay method. U-87 MG cells were treated with two inhibitors at a fixed ratio for 48 h. **d**-**e** U-87 MG cells were treated with PIK-75 (0.1 μM), TGX-221 (20 μM) or CAL-101 (10 μM) alone and combined with SP600125 (20 μM) for 24, 48, 72 and 96 h respectively. DMSO was used as a carrier control. Antagonistic effect was found in the combination of PIK-75 and SP600125, whereas SP600125 significantly synergized with TGX-221 or CAL-101 (*n* = 3; *p* values were determined by Two-way ANOVA and Post Hoc multiple comparison Tukey HSD test. ***: *p* <0.001). **g**-**i** U-87 MG cells were treated with PIK-75, TGX-221 or CAL-101 alone and combined with SP600125 for 3 h. Phosphorylation level of Akt was also downregulated by SP600125. Combination of TGX-211 and SP600125, as well as CAL-101 and SP600125 exhibited higher inhibition of Akt phosphorylation at Thr308

To evaluate the combination effects of isoform-selective PI3K inhibitors and SP600125 on glioblastoma cell proliferation U-87 MG cells were simultaneously treated with the inhibitors alone or in combination for 48 h. FA-CI plots were generated to evaluate the combination effect (Fig. 2a-c). The combination of PIK-75 and SP600125 showed antagonistic effects on U-87 MG cell proliferation with CI >1.1, whereas TGX-221/CAL-101 and SP600125 exhibited a moderate synergistic effect with CI <0.9 (Table 1,

**Table 1:** Combination index (CI) for cell viability in GBM cell line U-87 MG treated with SP600125 and PIK-75, TGX-221 or CAL-101 (*n* = 3)

PIK-75 (μM)	TGX-221 (μM)	CAL-101 (μM)	SP600125 (μM)	CI ^a^		
				PIK-75 + SP600125	TGX-221 + SP600125	CAL-101 + SP600125
0.01	2	1	2	1.333 ± 0.169	1.252 ± 0.040	1.614 ± 0.169
0.025	5	2.5	5	1.645 ± 0.082	0.907 ± 0.005	1.263 ± 0.301
0.05	10	5	10	1.595 ± 0.061	0.837 ± 0.001	0.911 ± 0.107
0.1	20	10	20	1.568 ± 0.047	0.855 ± 0.055	0.809 ± 0.018
0.2	40	20	40	2.104 ± 0.103	1.009 ± 0.125	0.700 ± 0.049

^a^Data were presented as mean ± S.E.M. CI <0.9 indicates synergistic effects; CI >1.1 indicates antagonistic effects; CI between 0.9 and 1.1 indicates additive effects

Additional file 1: Table S3). Interestingly too low or too high concentration of TGX-221 did not synergize with SP600125, indicating that the combination effect of TGX-221 and SP600125 may be dose-dependent (Table 1).

According to the findings from Western blotting and combination treatment we chose PIK-75 (0.1 μM), TGX-221 (20 μM), CAL-101 (10 μM) and SP600125 (20 μM) to conduct the subsequent experiments on glioblastoma cell migration and invasion. U-87 MG cells treated with inhibitors at these concentrations showed less cytotoxicity but significant inhibition of Akt or JNK signaling. Further, combination of TGX-221 and SP600125, as well as CAL-101 and SP600125 displayed synergistic effects at these concentrations. To investigate whether the combination treatment was time-dependent, U-87 MG cells were treated with PIK-75, TGX-221, or CAL-101 combined with SP600125 for 24, 48, 72 and 96 h. The inhibitory effects of drug combinations (TGX-221 and SP600125, CAL-101 and SP600125) were time-dependent, and SP600125 potentiated the inhibitory effect of TGX-221 or CAL-101 at 24, 48, 72 and 96 h (Fig. 2e-f). PIK-75 or SP600125 alone significantly inhibited cell viability in a time-dependent manner. However, combined treatment of PIK-75 and SP600125 attenuated the inhibitory effect of PIK-75, being in accordance with the observation that PIK-75 and SP600125 were antagonistic (Fig. 2d). Taken together, TGX-221 or CAL-101 synergized with SP600125 on glioblastoma cell proliferation inhibition in dose- and time- dependent manners, whereas combination of PIK-75 and SP600125 showed an antagonistic effect. Similar results were also found in U-373 MG cells, suggesting that the combination effect may not be cell line-specific (Additional file 1: Figure S3).

Western blotting was carried out to investigate the effects of drug combination on Akt and JNK signaling. It was found that JNK inhibitor not only inhibited c-Jun phosphorylation but also attenuated Akt phosphorylation at Thr308 (Fig. 2g-i). More importantly, combination of TGX-221/CAL-101 and SP600125 had higher inhibition of Akt phosphorylation at Thr308, but not at Ser473 and c-Jun phosphorylation (Fig. 2h, i). No significant change of Akt and c-Jun phosphorylation levels was observed in the combination of PIK-75 and SP600125 (Fig. 2g). Interestingly, inhibition of p110β remarkably suppressed c-Jun phosphorylation, but no further suppression was observed when JNK was also inhibited (Fig. 2h). This suggested that p110β and JNK may act on c-Jun via the same pathway.

Additionally experiments were also conducted in normal human astrocytes to evaluate the combination effects on normal brain cells. Treatments of PIK-75, TGX-221, CAL-101 and SP600125 alone, as well as their combination showed lower cytotoxicities to astrocytes than U-87 MG cells (Additional file 1: Figure S3).

### Inhibition of JNK potentiates the inhibitory effect of p110β/δ inhibitor on glioblastoma cell migration but not invasion

**Fig. 3:**
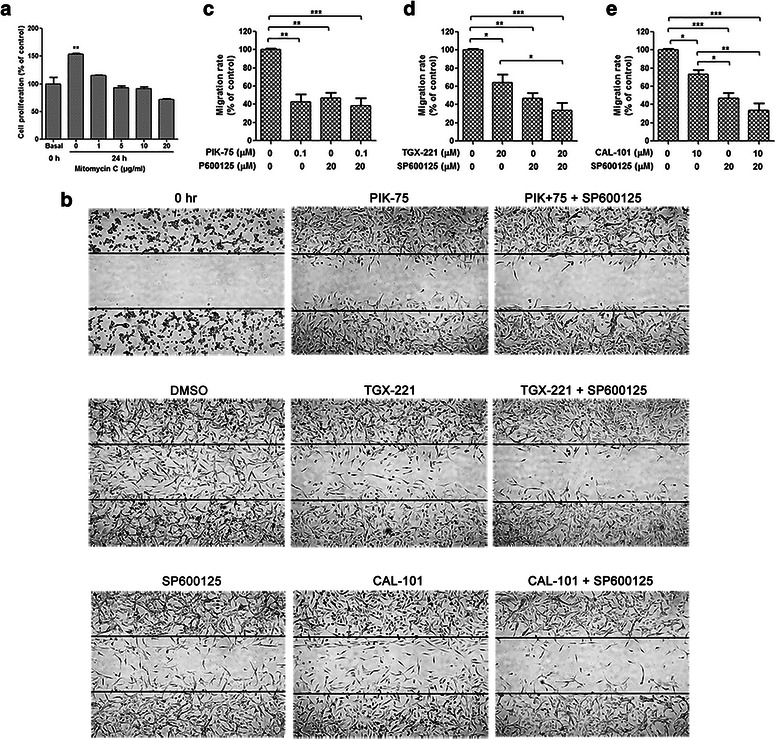
SP600125 potentiated inhibitory effects of isoform-selective PI3K inhibitors on glioblastoma cell migration. **a** Number of viable U-87 MG cells was counted using Vi-Cell Cell Viability Analyzer (Beckman Coulter) before and after treatment of mitomycin C for 24 h. Cell proliferation was inhibited by mitomycin C at 5 or 10 μg/mL without impairing viability. **b** Wound healing in U-87 MG cells treated with PIK-75 (0.1 μM), TGX-221 (20 μM) or CAL-101 (10 μM) alone and combined with SP600125 (20 μM) for 24 h. Cells were pretreated with 5 μg/mL of mitomycin C for 1 h. The lines indicate the edge of wound generated before drug treatment (0 h). Photographs were obtained at 50× magnification. **c**-**e** Migration rate was analyzed and expressed as the number of cells migrating into the original wounds. U-87 MG cell migration was blocked by PIK-75, TGX-221, CAL-101 and SP600125 alone. Inhibition of migration rate was reinforced by the combined treatment of TGX-221 and SP600125, as well as CAL-101 and SP600125 (*n* = 3; *p* values were determined by One-way ANOVA and Post Hoc multiple comparison Tukey HSD test. *: *p* <0.05; **: *p* <0.01; ***: *p* <0.001)

To investigate the combination effects of isoform-selective PI3K inhibitors and SP600125 on glioblastoma cell motility wound was generated in U-87 MG cells which were then treated with PIK-75 (0.1 μM), TGX-221 (20 μM), or CAL-101 (10 μM) combined with SP600125 (20 μM) for 24 h. In order to exclude the effect of cell proliferation, cells were incubated with mitomycin C prior to wound production and inhibitor treatment. It was found that mitomycin C at 5–10 μg/mL effectively inhibited glioblastoma cell growth without decreasing the live cell number (Fig. 3a). Therefore, 5 μg/mL was considered as the optimal concentration of mitomycin C to block glioblastoma cell proliferation. As shown in Fig. 3, PIK-75, TGX-221, CAL-101 and SP600125 were capable of preventing U-87 MG cells migrating into the wound to different extent. The capacity of blocking migration is roughly ranked as followed: PIK-75 ≈ SP600125 > TGX-221 > CAL-101. Combination of TGX-221 or CAL-101 combined with SP600125 potentiated the inhibitory effects on glioblastoma cell migration, whereas PIK-75 and SP600125 did not (Fig. 3e-g). Similar inhibitory effects were also observed in U-373 MG cells, in spite of the fact that only PIK-75 was sufficient to block the migration of these cells (Additional file 1: Figure S4).

**Fig. 4:**
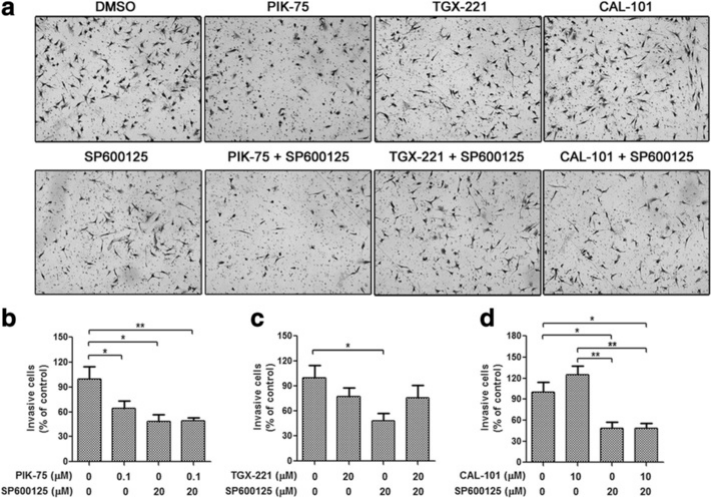
No synergistic effect on glioblastoma cell invasion was observed in the combination of isoform-selective PI3K inhibitors and SP600125. Boyden chamber invasion assay of U-87 MG cells treated with PIK-75 (0.1 μM), TGX-221 (20 μM) or CAL-101 (10 μM) alone and combined with SP600125 (20 μM) for 24 h. **a** Representative photographs showing the invasive cells that had passed through matrigel to the lower surface of the membrane at 100× magnification. Invaded cells from 5 representative fields were counted. **b**-**d** Number of invasive cells was significantly decreased by PIK-75 and SP600125, and all the combinations did not enhanced the inhibitory effect (*n* = 3; *p* values were determined by One-way ANOVA and Post Hoc multiple comparison Tukey HSD test. *: *p* <0.05; **: *p* <0.01)

To analyze the effects of isoform-selectivePI3K inhibitors in combination with SP600125 on glioblastoma cell invasion Boyden chamber invasion assay was performed on U-87 MG and U-373 MG cells. Number of invasive cells was markedly decreased by PIK-75 and SP600125, whereas neither TGX-221 nor CAL-101 inhibited glioblastoma cell invasion (Fig. 4). No synergistic effect was found with combination treatment on cell invasion (Fig. 4 and Additional file 1: Figure S5).

### Combined inhibition of p110β/δ and JNK restrains lamellipodia and membrane ruffles formation through decreased FAK or zyxin activation

**Fig. 5:**
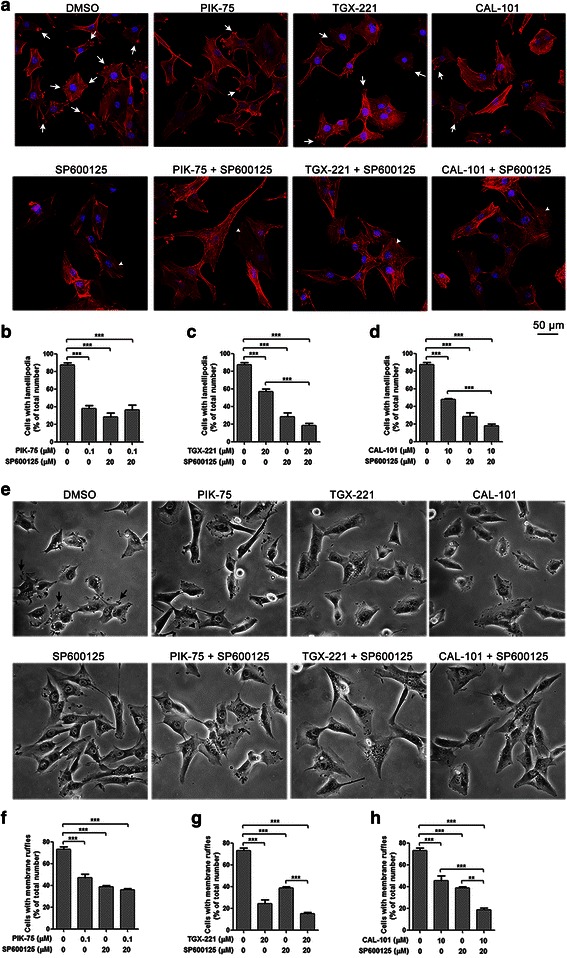
SP600125 potentiated inhibitory effects of isoform-selective PI3K inhibitors on lamellipodia and membrane ruffles formation. **a** Immunocytochemistry of U-87 MG cells stained with Alexa Fluor 594-conjugated phalloidin (red) after treatment with PIK-75 (0.1 μM), TGX-221 (20 μM) or CAL-101 (10 μM) alone and combined with SP600125 (20 μM) for 3 h. Lamellipodia (white arrow) were decreased after inhibition of p110α, p110β, p110δ and JNK, while stress fibers (arrowhead) were widely developed at the presence of SP600125. Bar = 50 μm. **b**-**d** Cells with lamellipodia were counted and data were expressed as the ratio of number of cells with lamellipodia over total cell number. SP600125 potentiated the inhibitory effect of TGX-221 and CAL-101 on lamellipodia formation (*n* = 3; *p* values were determined by One-way ANOVA and Post Hoc multiple comparison Tukey HSD test. ***: *p* <0.001). **e** Phase contrast microscopy of U-87 MG cells after treatment with PIK-75, TGX-221 or CAL-101 alone and combined with SP600125 for 3 h. Cells with membrane ruffles (black arrow) were reduced by the treatment of isoform-selective PI3K inhibitors and SP600125. Photographs were obtained at 200× magnification. **f**-**h** Cells with membrane ruffles were counted and data were expressed as the ratio of number of cells with membrane ruffles over total cell number. SP600125 also enhanced the inhibitory effect of TGX-221 and CAL-101 on membrane ruffles formation (*n* = 3; *p* values were determined by One-way ANOVA and Post Hoc multiple comparison Tukey HSD test. **: *p* <0.01; ***: *p* <0.001)

Actin remodeling which includes formation of lamellipodia and membrane ruffles is essential to cell migration. Therefore we assessed the effects of isoform-selective PI3K inhibitors and JNK inhibitor on lamellipodia and membrane ruffles formation in glioblastoma cells by labeling actin filaments after 3-h drug treatment. Number of U-87 MG cells with lamellipodia and membrane ruffles was significantly decreased after treatment with inhibitors alone (Fig. 5). Interestingly, among the isoform-selective PI3K inhibitors, inhibition of membrane ruffles formation was the highest upon the treatment of the p110β inhibitor, whereas lamellipodia formation was most significantly impaired when treated with the p110α inhibitor, suggesting that p110β may be required for membrane ruffles formation, while p110α is essential for lamellipodia formation. Combination of TGX-221/CAL-101 and SP600125 potentiated the inhibitory effects, whereas PIK-75 and SP600125 did not.

**Fig. 6:**
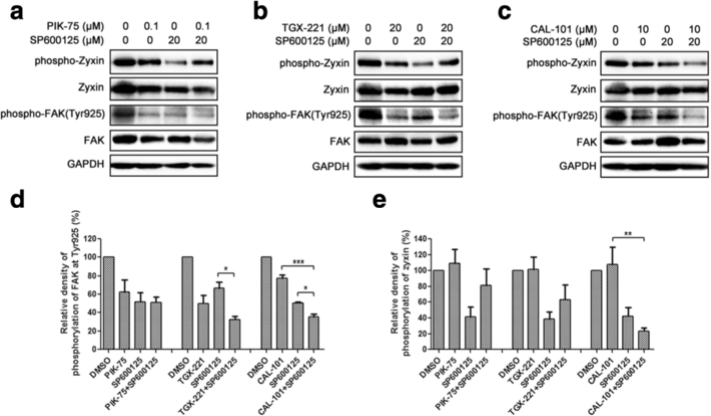
Isoform-selective PI3K inhibitors and SP600125 affected focal adhesion and cytoskeleton-related signaling. **a**-**c** U-87 MG cells were treated with PIK-75 (0.1 μM), TGX-221 (20 μM) or CAL-101 (10 μM) alone and combined with SP600125 (20 μM) for 24 h. Whole cell lysates were separated by SDA-PAGE, and the phosphorylated and total protein levels were determined by Western blotting using antibodies against the corresponding phosphorylated and total proteins. Expression of GAPDH was served as a loading control. Data were representative of three independent experiments. Density of FAK **d** and zyxin **e** phosphorylation levels were relative to total FAK and zyxin protein expression levels. Combination of TGX-221 and SP600125 showed a higher inhibition of FAK phosphorylation, while combination of CAL-101 and SP600125 displayed higher inhibition of FAK and zyxin phosphorylation (*n* = 3; *p* values were determined by One-way ANOVA and Post Hoc multiple comparison Tukey HSD test. *: *p* <0.05; **: *p* <0.01)

To elucidate the molecular mechanism contributing to the synergistic effect of isoform-selective PI3K inhibitors and JNK inhibitor on cell migration activation of focal adhesion and cytoskeleton-related signaling network were investigated. After 24-h drug treatment, phosphorylation level of FAK at Tyr925 in U-87 MG cells was decreased upon inhibition of p110 isoforms and JNK, whereas the phosphorylation level of zyxin was only reduced by inhibition of JNK (Fig. 6a-c). Moreover, combination of TGX-221 and SP600125 showed lower FAK phosphorylation level, while combination of CAL-101 and SP600125 displayed lower phosphorylation levels of both zyxin and FAK (Fig. 6b-e). Thus, isoform-selective PI3K inhibitors impeded glioblastoma cell migration through blockade of FAK activation, and reduced FAK or zyxin activation contributed to the synergistic inhibitory effects on glioblastoma cell migration.

### Combined inhibition of p110β and JNK suppresses xenograft tumor growth in vivo

**Fig. 7:**
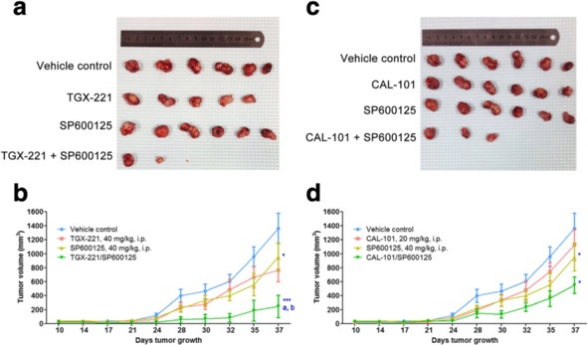
Combined inhibition of p110β and JNK exerted synergism to suppress xenograft tumor growth in vivo. Human glioblastoma U-87 MG cells (5 × 10^6^ cells) were subcutaneously injected into the Balb/C nude mice. Nine days after inoculation, mice were intraperitoneally (i.p.) injected once daily for 7 days with vehicle, TGX-221 (40 mg/kg) and CAL-101 (20 mg/kg) alone, or in combination with SP600125 (40 mg/kg). Measurement of tumor volumes started on the day of the first administration. **a**, **c** Representative subcutaneous tumor xenografts from mice sacrificed after 37-day post-administration. **b**, **d** Summary data of tumor volumes from 10-day post-administration to the end of the experiment. (*n* = 6; *p* values were determined by One-way repeated measures ANOVA and Post Hoc multiple comparison Tukey HSD test. *: *p* <0.05; ***: *p* <0.001 compared with vehicle control; a: *p* <0.05 compared with PI3K inhibitor; b: *p* <0.05 compared with SP600125)

The in vitro synergistic effects of p110β/δ inhibitors and SP600125 on glioblastoma cell proliferation and migration suggest that they may have similar anti-tumor activity in vivo. To evaluate the combination effect of p110β/δ and JNK inhibition on tumor growth Balb/C nude mice bearing U-87 MG glioblastoma xenograft were treated with vehicle, TGX-221 (40 mg/kg) and CAL-101 (20 mg/kg) alone, or in combination with SP600125 (40 mg/kg). Only SP600125 significantly suppressed U-87 MG xenograft tumor growth after 24-day post-administration, whereas TGX-221 or CAL-101 slightly decreased tumor volumes (Fig. 7). Synergistic inhibitory effect on tumor growth was observed in the combination treatment of TGX-221 and SP600125, being consistent with the in vitro study (Fig. 7a-b). Although combination of CAL-101 and SP600125 effectively reduced tumor volume, there is no significant difference between combination treatment and single inhibitor alone (Fig. 7c-d). However, about half of mice with combination treatment died on the next day of intraperitoneal injection, while the remaining mice with the same treatment did not show any adverse reaction until sacrifice, suggesting that the death of mice may be correlated with over-injection, and the therapeutic tolerance of combination treatments was acceptable.

## Discussion

The roles of class I_A_ PI3K isoforms in the pathological processes of glioblastoma, and the crosstalk between PI3K and JNK are complicated. In this study, we employed three selective inhibitors PIK-75, TGX-221 and CAL-101, which targets p110α, p110β, and p110δ isoform respectively, to investigate the effects of these catalytic subunits on glioblastoma cells. We demonstrated that these PI3K isoforms played distinct roles on proliferation, migration and invasion of glioblastoma cells, even though their respective inhibitors inhibited Akt phosphorylation at both Ser473 and Thr308. Inhibition of p110α was sufficient to suppress glioblastoma cell viability, migration and invasion, whereas inhibition of p110β only blocked cell migration, and inhibition of p110δ moderately impeded cell proliferation and migration. Our data indicated that p110α is of more importance to glioblastoma cell viability and motility than p110β and p110δ.

Accumulating evidence shows that the p110 isoforms exert distinct roles in Akt phosphorylation and other pathological processes [16, 17]. The p110α and p110β display different preferences to RTK activation, but p110β is also activated by GPCRs [18, 19]. Selective gene knockdown and isoform-selective inhibitors are helpful to investigate the individual role of class I_A_ PI3K catalytic isoforms. The p110α isoform is required for tumor cell proliferation, migration and invasion, whereas p110β is essential to cell survival and tumorigenesis [20–23]. Knockdown of *PIK3CA* (encoding p110α) significantly inhibits cell viability and motility in medulloblastoma and glioblastoma cells [20, 21]. The p110α isoform is also vital to invasion of breast cancer cells through mediating invadopodia formation [24]. In addition, tumor cell growth is effectively suppressed in vitro and in vivo by using the p110α isoform-selective inhibitors A66, BYL719 or PIK-75, while apoptosis and cell cycle arrest are promoted [25–28]. Our findings are in agreement with these studies. Knockdown of *PIK3CB* (encoding p110β) suppresses cell proliferation and induces apoptosis in ovarian cancer and glioblastoma in vitro and in vivo, suggesting p110β plays important roles in cell growth [22, 29]. However, selective p110β inhibitor TGX-221 does not affect cell proliferation or apoptosis, but impedes migration in glioblastoma cells [30]. These distinct outcomes between selective inhibitor and gene knockout may be caused by kinase-independent function of p110β [31]. In addition, targeting p110δ suppresses cell growth, migration and invasion in neuroblastoma and glioblastoma cells [32, 33]. Our previous study shows that the p110δ-selective inhibitor IC87114 only suppresses cell migration without affecting the invasive capacity [33]. In this study, similar results were also found in glioblastoma cells using inhibitor CAL-101 which had higher selectivity to p110δ. Similar to our findings, Holand et al. found that PIK-75 significantly inhibited T98G glioblastoma cell proliferation and colony formation, while TGX-221 and IC87114 did not show the inhibitory effects [30]. Taken together, consistent results were obtained in glioblastoma cell lines U-87 MG, U-373 MG and T98G with different genetic background, suggesting that p110α is most essential among three class I_A_ PI3K catalytic isoforms, which may be due to its predominant role in RTK-mediated PI3K signaling. In contrast, inhibition of p110β and p110δ exerts minor influence on cellular functions of glioblastoma cells, which may be limited by the constitutive activation of p110α [34].

The ATP-competitive JNK inhibitor SP600125 was sufficient to inhibit cell viability, migration and invasion of glioblastoma cells through decreasing the phosphorylation levels of both c-Jun and Akt. Activated JNK can translocate into the nucleus and phosphorylate a variety of transcription factors such as c-Jun, which is a component of transcription factor activator protein-1 (AP-1). AP-1 is able to regulate the transcriptional activity of phosphoinositide-dependent kinase 1 (PDK1) and the activation of its downstream target Akt, suggesting that JNK regulates Akt activity through JNK/c-Jun/PDK1/Akt axis [35]. Interestingly, we also found that only TGX-221 was able to decrease c-Jun phosphorylation level, which was in accordance with the constitutive activation of p110β contributing to the activation of JNK [10]. Our data therefore suggest that p110β, not p110α nor p110δ, may be an upstream regulator of JNK signaling, possibly because they are both activated by GPCRs.

Combination therapy targeting PI3K/Akt pathway and Ras-mediated MAPK pathway may exert synergism for cancer treatment [36, 37]. Evidence shows that combined inhibition of p110δ and mitogen-extracellular activated protein kinase (MEK) has synergistic cytotoxicity in human acute myeloid leukemia progenitors [38]. Combination of MEK1/2 and PI3K/mTOR inhibitors potentiates the inhibitory effects on cell proliferation and angiogenesis in non-small cell lung carcinoma (NSCLC) [39]. Concurrent expression of dominant-negative mitogen-activated protein kinase kinase 4 (MKK4) and PI3K p85α isoform is also synergistic to induce apoptosis in lung cancer [40]. This kind of synergism is also noted in this study in which combined inhibition of p110β/δ and JNK displayed synergistic inhibitory effects on glioblastoma cell proliferation and migration via blockade of Akt, zyxin and FAK activation. Formation of lamellipodia and membrane ruffles involved in cell migration was also suppressed. However, only PIK-75 and SP600125 suppressed glioblastoma cell invasion, and all the combinations had no synergistic effects, suggesting that the crosstalk between PI3K and JNK in cell invasion is limited. Although the p110α isoform plays a dominant role in the activation of PI3K/Akt signaling, p110β modulates PI3K activity in a competitive manner by competing against p110α for RTK binding [41]. Evidence shows that genetic deletion of *PI3KCA* suppresses tumor formation of breast cancer, whereas loss of *PIK3CB* leads to mammary gland hyperplasia and tumorigenesis, accompanied by increased PI3K activity and Akt phosphorylation level [41]. In addition, overexpression of p110β blocks the EGF-induced migration in breast cancer cells, suggesting abundant p110β may replace p110α to interact with EGFR and results in deficiency of active p110α [42]. Since p110α plays a dominant role in RTK-mediated Akt signaling, inhibition of p110α by PIK-75 may lead to activation of p110β due to a compensatory effect induced by GPCR activation, and then p110β may activate JNK pathway to escape from p110α inhibition. We also found that phosphorylation level of c-Jun was elevated by treatment of PIK-75, indicating that inhibition of p110α may promote JNK activity through activation of p110β/GPCR/JNK signaling axis. It may explain why combined inhibition of p110α and JNK was antagonism in our study. However, since PIK-75 displays an unexpected inhibition on DNA-PK, and BYL-719 exhibits better pharmaceutical properties, selectivity and sensitivity to p110α than PIK-75, BYL-719 will be employed in the future in vitro and in vivo study to confirm our findings.

Being consistent with the in vitro study, synergistic inhibitory effect of combination between TGX-221 and SP600125 on glioblastoma cell U-87 MG xenograft tumor growth was also observed. However, siRNA-mediated gene knockdown and pharmacological inhibition of the same molecule may not generate the same effect. To confirm our findings and figure out which JNK isoform participates in the crosstalk with PI3K p110β, siRNAs targeting JNK1, JNK2 and JNK3 respectively will be combined with p110β inhibitor in the future study. Compared with vehicle control, treatment of CAL-101 did not decrease xenograft tumor growth, indicating that p110δ plays a minor role in glioblastoma growth. Although the combination of CAL-101 and SP600125 remarkably reduced tumor growth, there was no significant difference between this combination and single inhibitor alone, suggesting that higher dose of CAL-101 may be employed to augment the combination effect.

Cell migration is a complicated biological process including actin polymerization, protrusion formation, adhesion complexes formation, turnover of focal adhesion, and retraction at the cell rear. A variety of cytoskeletal structures including lamellipodia, filapodia, focal adhesion, stress fibers, and membrane ruffles are essential to a motile cell, providing adhesion sites and contractile forces for cell movement. Focal adhesion complexes consisting of integrin, paxillin, talin, zyxin and FAK are formed at the edge of lamellipodia to provide attachment of cell protrusion to the extracellular matrix [43]. Zyxin and FAK are known as focal adhesion proteins mediating cell-matrix adhesion and actin polymerization. Zyxin recruits Enabled/vasodilator-stimulated phospho-protein (Ena/VASP) through N-terminal ActA proline-rich domain to regulate actin filament assembly, and facilitates focal adhesion by interacting with specific proteins through C-terminal LIM domains [44]. The interaction of LIM domains and adaptors required phosphorylation of zyxin at Ser142 [45]. Evidence shows that knockdown or inhibition of zyxin leads to blockade of cell spreading and migration [46]. Here, we demonstrated that SP600125 markedly decreased zyxin phosphorylation level, whereas none of the isoform-selective PI3K inhibitors did so, suggesting that the impact of JNK and PI3K inhibitors on downstream signaling is different. Moreover, combination of CAL-101 and SP600125 synergistically reduced zyxin phosphorylation level than on their own. In addition, activation of FAK is involved in cell adhesion, migration and invasion. Autophosphorylation of FAK at Tyr397 leads to recruitment of PI3K p85 subunit and phosphorylation of FAK at Tyr925, and subsequently triggers the Ras-dependent activation of MAPK pathway. FAK phosphorylation status at Tyr925 is crucial to cell migration and focal adhesion turnover. Evidence reveals that expression of nonphosphorylatable FAK at Tyr925 in FAK^−/−^ mouse embryonic fibroblasts (MEFs) reduces phosphorylation of paxillin and activates Rac1, leading to stabilization of focal adhesion, inhibition of cell protrusion and migration [47]. In this study, isoform-selective PI3K inhibitors and SP600125 alone suppressed glioblastoma cell migration through inhibition of FAK phosphorylation at Tyr925. Combination of TGX-221 and SP600125, as well as CAL-101 and SP600125 also potentiated the inhibitory effects on FAK phosphorylation.

## Conclusions

In summary, the isoform-selective PI3K inhibitors displayed distinct effects on glioblastoma cell proliferation, migration and invasion in vitro. Combined inhibition of p110β and JNK exhibited synergistic effects on suppressing glioblastoma cell proliferation and migration in vitro and xenograft tumor growth in vivo through decreasing Akt and FAK phosphorylation, resulting in the blockade of lamellipodia and membrane ruffles formation. More importantly, these inhibitors, either alone or in combination, were much less toxic to astrocytes than glioblastoma cells in vitro. Preclinical study also suggested that the toxicity was tolerable in mice. Taken together, combination therapy via concurrent inhibition of PI3K and JNK may be a promising approach for glioblastoma multiforme treatment.

## Additional file

Additional file 1: Supplementary Table 1. Characteristics of isoform-selective PI3K inhibitors. **Supplementary Table 2.** Characteristics of JNK inhibitor SP600125. **Supplementary Table 3.** Combination index (CI) for cell viability in GBM cell line U-87 MG in the combination of PIK-75 and SP600125 (*n* = 3). **Supplementary Fig. 1**: Half-maximal inhibitory concentration (IC_50_) values of PIK-75, TGX-221, CAL-101 and SP600125 on glioblastoma cell line U87-MG. **Supplementary Fig. 2.** Effects of PIK-75, TGX-221, CAL-101 and SP600125 on cell viability of glioblastoma cell line U-373 MG. **Supplementary Fig. 3.** Combination effects of isoform-selective PI3K inhibitors and SP600125 on proliferation of glioblastoma cells and normal human astrocytes. **Supplementary Fig. 4.** Combination effects of isoform-selective PI3K inhibitors and SP600125 on migration of U-373 MG glioblastoma cells. **Supplementary Fig. 5.** Combination effects of isoform-selective PI3K inhibitors and SP600125 on invasion of U-373 MG glioblastoma cells. **Supplementary Fig. 6.** Combination effects of isoform-selective PI3K inhibitors and SP600125 on glioblastoma U-87 MG cell apoptosis. (DOC 3768 kb)

## Abbreviations

AP-1: transcription factor activator protein-1
BSA: bovine serum albumin
CI: combination index
DMSO: dimethyl sulfoxide
EGFR: epidermal growth factor receptor
Ena/VASP: enabled/vasodilator-stimulated phospho-protein
FA: fraction affected
FAK: focal adhesion kinase
FBS: fetal bovine serum
GBM: glioblastoma multiforme
GPCR: G protein–coupled receptor
JNK c-Jun: N-terminal kinase
MEF: mouse embryonic fibroblast
MEK: mitogen-extracellular activated protein kinase
MKK4: mitogen-activated protein kinase kinase 4
MTT: 3-(4,5-Dimethylthiazol-2-yl)-2,5-diphenyltetrazolium bromide
NSCLC: non-small cell lung carcinoma
PDK1: phosphoinositide-dependent kinase 1
PI3K: phosphatidylinositol 3-kinase
RTK: receptor tyrosine kinase
