# Supplementary material for: PI3K p110β isoform synergizes with JNK in the regulation of glioblastoma cell proliferation and migration through Akt and FAK inhibition
Source: J Exp Clin Cancer Res. 2016 May 12;35:78. doi: 10.1186/s13046-016-0356-5 (PMC4866398; doi:10.1186/s13046-016-0356-5)
Supplement: Additional file 1: Supplementary Table 1. — Characteristics of isoform-selective PI3K inhibitors. Supplementary Table 2. Characteristics of JNK inhibitor SP600125. Supplementary Table 3. Combination index (CI) for cell viability in GBM cell line U-87 MG in the combination of PIK-75 and SP600125 (n = 3). Supplementary Fig. 1: Half-maximal inhibitory concentration (IC50) values of PIK-75, TGX-221, CAL-101 and SP600125 on glioblastoma cell line U87-MG. Supplementary Fig. 2. Effects of PIK-75, TGX-221, CAL-101 and SP600125 on cell viability of glioblastoma cell line U-373 MG. Supplementary Fig. 3. Combination effects of isoform-selective PI3K inhibitors and SP600125 on proliferation of glioblastoma cells and normal human astrocytes. Supplementary Fig. 4. Combination effects of isoform-selective PI3K inhibitors and SP600125 on migration of U-373 MG glioblastoma cells. Supplementary Fig. 5. Combination effects of isoform-selective PI3K inhibitors and SP600125 on invasion of U-373 MG glioblastoma cells. Supplementary Fig. 6. Combination effects of isoform-selective PI3K inhibitors and SP600125 on glioblastoma U-87 MG cell apoptosis. (DOC 3768 kb) [file 13046_2016_356_MOESM1_ESM.doc]

**Supplementary Tables and Figures**

**Supplementary Table 1. Characteristics** of isoform-selective PI3K inhibitors.

| **Compound** | | **IC_50_ (nM)** | | | | | | | | **Reference** |
| --- | --- | --- | --- | --- | --- | --- | --- | --- | --- | --- |
|  | **p110α** | | **p110β** | | **p110δ** | | **p110γ** | |  | |
| PIK-75 | | 5.8 | | 1300 | | 510 | | 76 | |  |
| TGX-221 | | 5000 | | 5 | | 211 | | > 3500 | |  |
| CAL-101 | | 820 | | 565 | | 2.5 | | 89 | |  |
| A66 | | 32 | | >12500 | | >1250 | | 3480 | |  |
| BYL719 | | 5 | | 1200 | | 290 | | 250 | |  |
| HS-173 | | 0.8 | | N/A | | N/A | | N/A | |  |

N/A, not available.

**Supplementary Table 2.** **Characteristics** of JNK inhibitor SP600125.

| **Compound** | **IC_50_ (nM)** | | | | | **Reference** |
| --- | --- | --- | --- | --- | --- | --- |
|  | **JNK1** | **JNK2** | **JNK3** | **MKK4** | **MKK7** |  |
| SP600125 | 40 | 40 | 90 | 400 | 5100 |  |

1. Zheng Z, Amran SI, Thompson PE, Jennings IG: **Isoform-selective inhibition of phosphoinositide 3-kinase: identification of a new region of nonconserved amino acids critical for p110alpha inhibition.** *Mol Pharmacol* 2011, **80:**657-664.

2. Jackson SP, Schoenwaelder SM, Goncalves I, Nesbitt WS, Yap CL, Wright CE, Kenche V, Anderson KE, Dopheide SM, Yuan Y, et al: **PI 3-kinase p110beta: a new target for antithrombotic therapy.** *Nat Med* 2005, **11:**507-514.

3. Lannutti BJ, Meadows SA, Herman SEM, Kashishian A, Steiner B, Johnson AJ, Byrd JC, Tyner JW, Loriaux MM, Deininger M, et al: **CAL-101, a p110  selective phosphatidylinositol-3-kinase inhibitor for the treatment of B-cell malignancies, inhibits PI3K signaling and cellular viability.** *Blood* 2010, **117:**591-594.

4. Jamieson S, Flanagan JU, Kolekar S, Buchanan C, Kendall JD, Lee WJ, Rewcastle GW, Denny WA, Singh R, Dickson J, et al: **A drug targeting only p110alpha can block phosphoinositide 3-kinase signalling and tumour growth in certain cell types.** *Biochem J* 2011, **438:**53-62.

5. Furet P, Guagnano V, Fairhurst RA, Imbach-Weese P, Bruce I, Knapp M, Fritsch C, Blasco F, Blanz J, Aichholz R, et al: **Discovery of NVP-BYL719 a potent and selective phosphatidylinositol-3 kinase alpha inhibitor selected for clinical evaluation.** *Bioorg Med Chem Lett* 2013, **23:**3741-3748.

6. Kim O, Jeong Y, Lee H, Hong SS, Hong S: **Design and synthesis of imidazopyridine analogues as inhibitors of phosphoinositide 3-kinase signaling and angiogenesis.** *J Med Chem* 2011, **54:**2455-2466.

7. Bennett BL, Sasaki DT, Murray BW, O'Leary EC, Sakata ST, Xu W, Leisten JC, Motiwala A, Pierce S, Satoh Y, et al: **SP600125, an anthrapyrazolone inhibitor of Jun N-terminal kinase.** *Proc Natl Acad Sci U S A* 2001, **98:**13681-13686.

**Supplementary Table 3. Combination index (CI) for cell viability in GBM cell line U-87 MG in the combination of PIK-75 and SP600125 (n=3)**

| **PIK-75 (μM)** | **SP600125 (μM)** | **CI** * |
| --- | --- | --- |
| 0.05 | 2 | 6.457 ± 2.157 |
| 0.125 | 5 | 2.164 ± 0.412 |
| 0.25 | 10 | 1.166 ± 0.176 |
| 0.5 | 20 | 1.369 ± 0.175 |
| 1 | 40 | 1.262 ± 0.369 |

* Data were presented as mean ± S.E.M. CI < 0.9 indicates synergistic effect; CI > 1.1 indicates antagonistic effect; CI between 0.9 and 1.1 indicates additive effect.


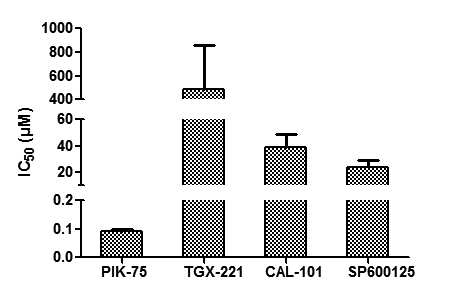


**Supplementary Figure 1: Half-maximal inhibitory concentration (IC_50_) values of PIK-75, TGX-221, CAL-101 and SP600125 on glioblastoma cell line U87-MG.** Cells were treated with PIK-75, TGX-221, CAL-101 or SP600125 at different concentrations for 48 hr. Growth inhibition by drug treatment was determined and IC_50_ values were calculated using Origin 9.0 software.


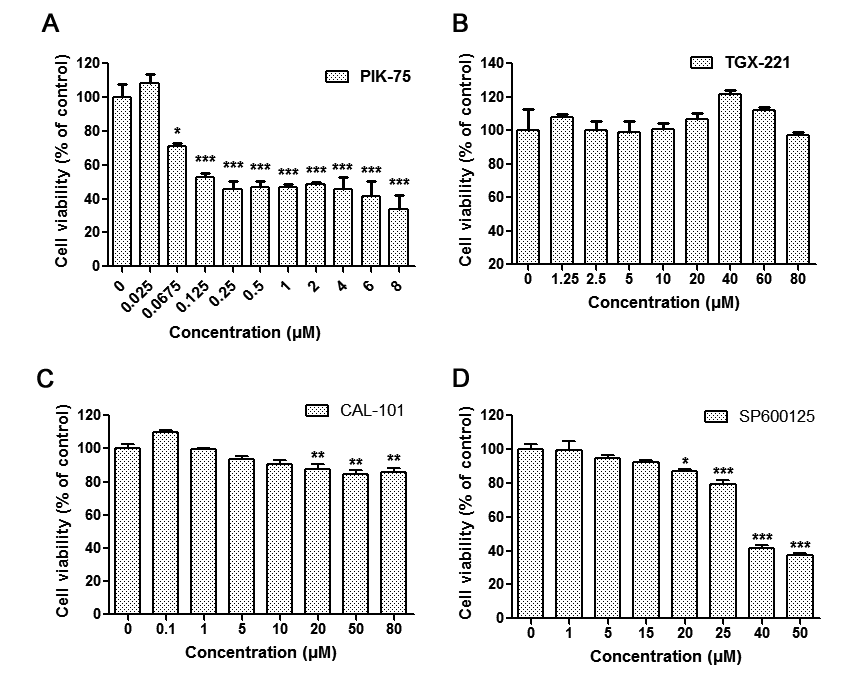


**Supplementary Figure 2: Effects of PIK-75, TGX-221, CAL-101 and SP600125 on cell viability of glioblastoma cell line U-373 MG.** U-373 MG cells were treated with PIK-75, TGX-221, CAL-101 or SP600125 respectively at different concentrations for 48 hr. DMSO was used as a carrier control. Cell viability of U-373 MG cells was significantly reduced when treated with PIK-75, CAL-101 and SP600125, whereas TGX-221 showed no significant effect (n=3; *p* values were determined by One-way ANOVA and Post Hoc multiple comparison Tukey HSD test. *: *p* <0.05; **: *p* <0.01; ***: *p* <0.001).


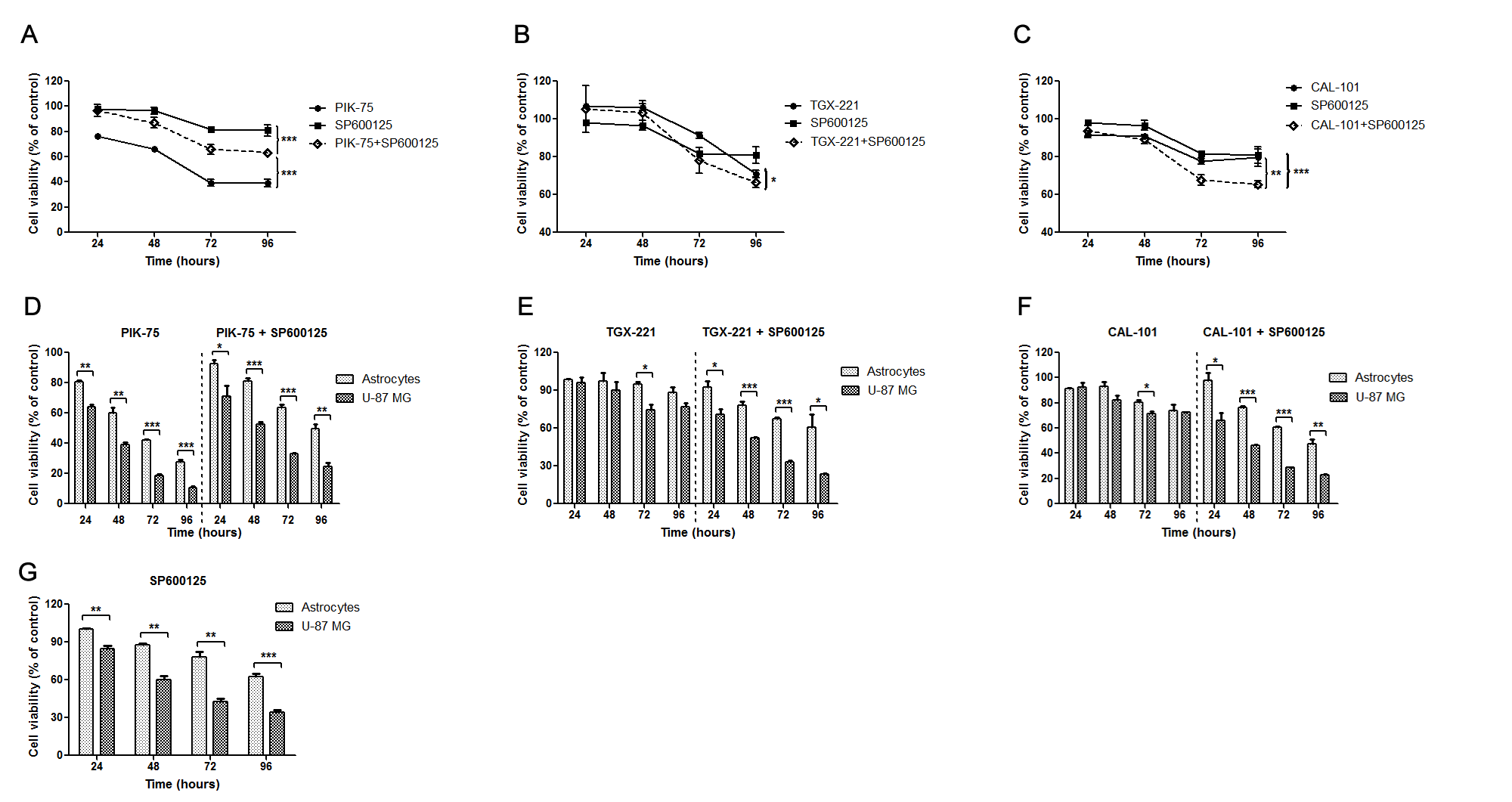


**Supplementary Figure 3: Combination effects of isoform-selective PI3K inhibitors and SP600125 on proliferation of glioblastoma cells and normal human astrocytes.** (A-C) U-373 MG cells were treated with PIK-75 (0.1 μM), TGX-221 (20 μM) or CAL-101 (10 μM) alone and combined with SP600125 (20 μM) for 24, 48, 72 and 96 hr respectively. DMSO was used as a carrier control. PIK-75 combined with SP600125 showed antagonistic effect, whereas SP600125 slightly synergized TGX-221 or CAL-101 at 72 and 96 hr (n=3; *p* values were determined by Two-way ANOVA and Post Hoc multiple comparison Tukey HSD test.*: *p* <0.05; **: *p* <0.01; ***: *p* <0.001). (D-G) Isoform-selective inhibitors and SP600125 displayed less cytotoxicities to normal human astrocytes. Glioblastoma cells U-87 MG and human astrocytes were treated with PIK-75 (0.1 μM), TGX-221 (20 μM) or CAL-101 (10 μM) alone and combined with SP600125 (20 μM) for 24, 48, 72 and 96 hr respectively. Compared with U-87 MG cells, astrocytes showed higher viabilities when treated with PIK-75, TGX-221 or CAL-101 alone and combined with SP600125 (n=3; *p* values were determined by independent sample *t* test.*: *p* <0.05; **: *p* <0.01; ***: *p* <0.001).


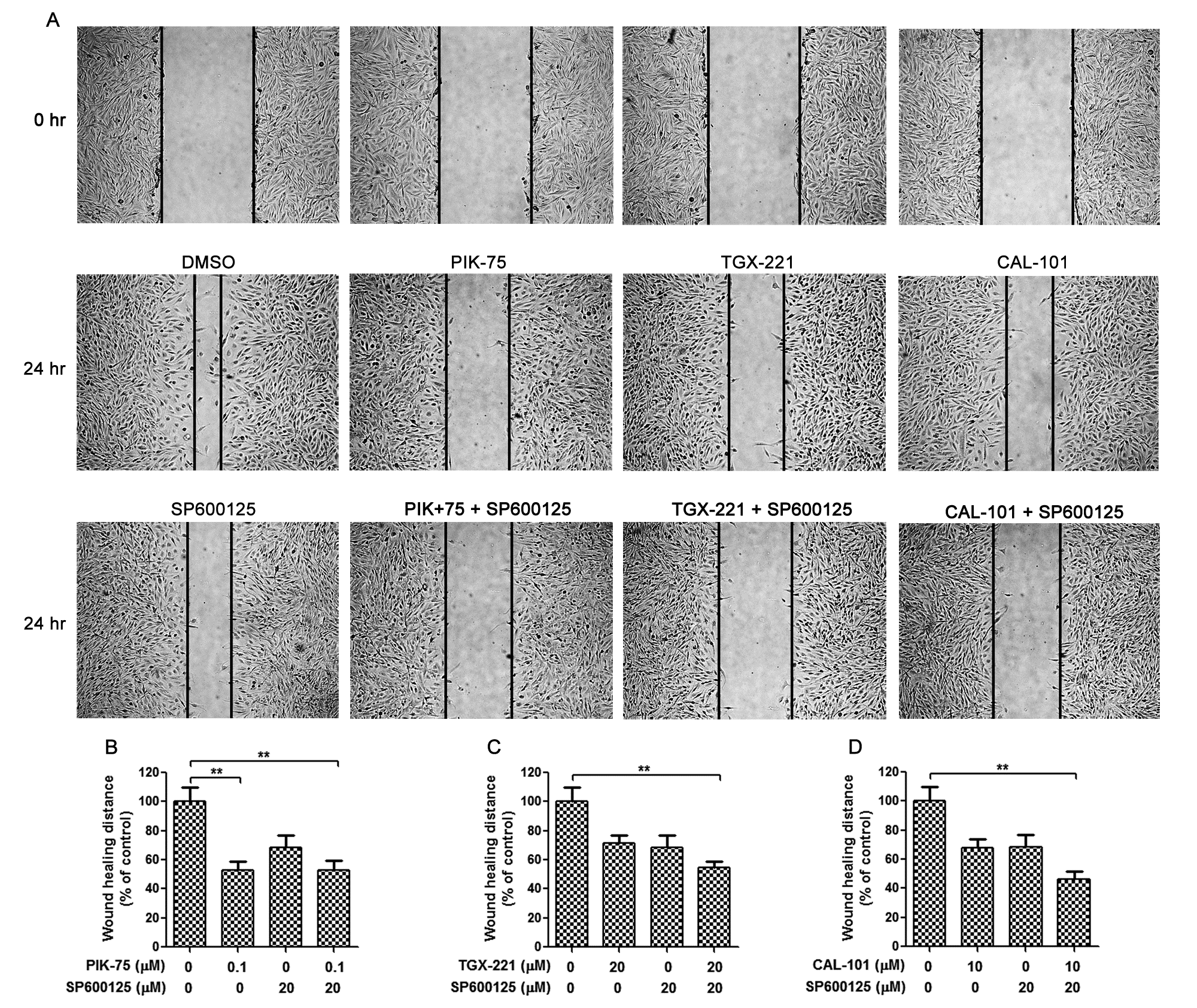


**Supplementary Figure 4: Combination effects of isoform-selective PI3K inhibitors and SP600125 on migration of U-373 MG glioblastoma cells.** (A) Wound healing ability of U-373 MG cells was tested when treated with PIK-75 (0.1 μM), TGX-221 (20 μM) or CAL-101 (10 μM) alone and combined with SP600125 (20 μM) for 24 hr. Cells were pretreated with 5 μg/mL of mitomycin C for 1 hr. The line indicates the edge of wound generated before (0 hr) or after (24 hr) drug treatment. Photographs were obtained at 50× magnification. (B-D) Wound healing distance (%) was analyzed and expressed as the migration distance of cells relative to that of DMSO control. PIK-75 alone was sufficient to block U-373 MG cell migration significantly, but combination of PIK-75 and SP600125 did not potentiate the inhibitory effect. However, cell migration was significantly suppressed by the combined treatment of TGX-221 and SP600125, as well as CAL-101 and SP600125, although these inhibitors alone had no significant inhibitory effect (n=3; *p* values were determined by One-way ANOVA and Post Hoc multiple comparison Tukey HSD test. **: *p* <0.01).


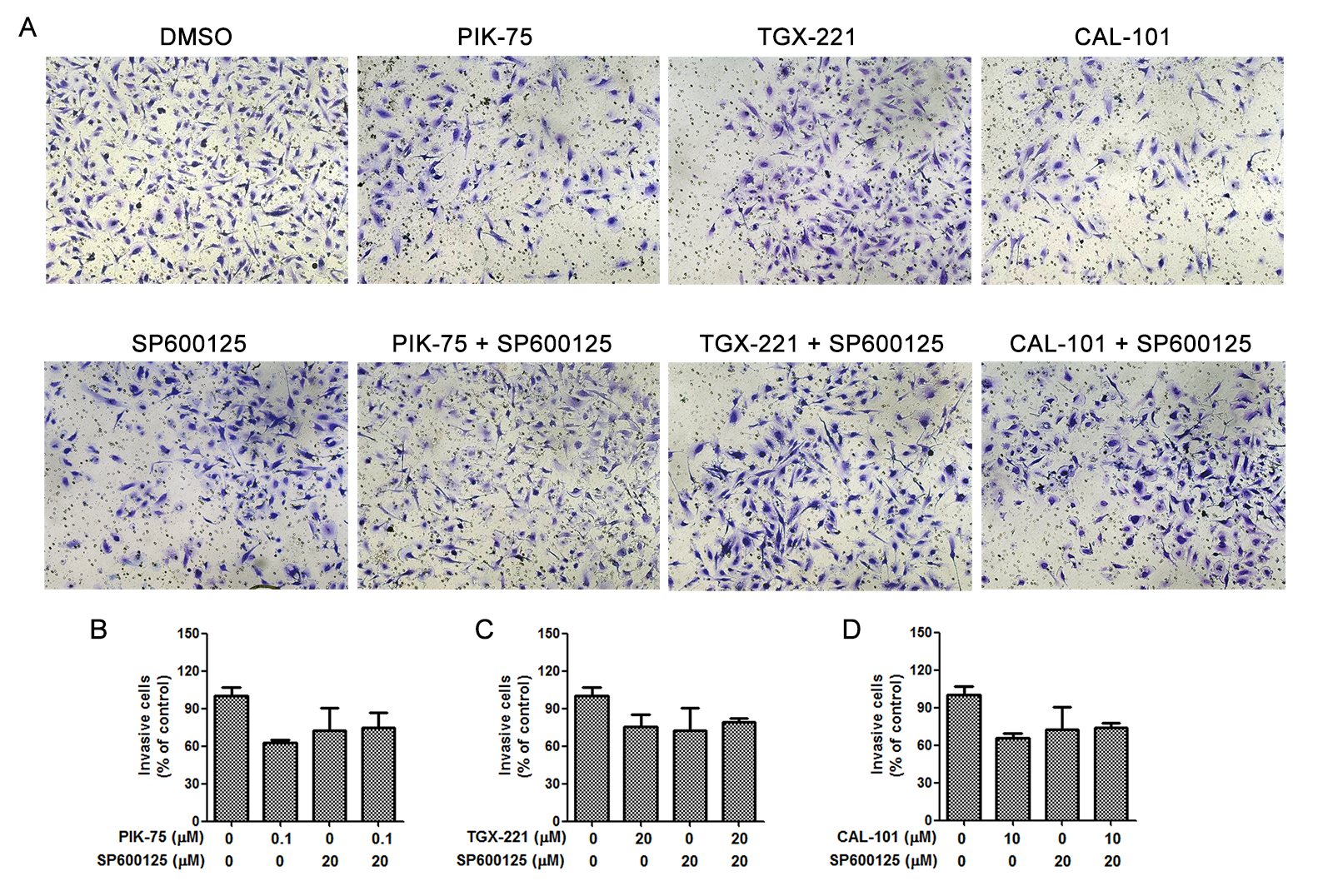


**Supplementary Figure 5: Combination effects of isoform-selective PI3K inhibitors and SP600125 on invasion of U-373 MG glioblastoma cells.** (A) Boyden chamber invasion assay of U-373 MG cells treated with PIK-75 (0.1 μM), TGX-221 (20 μM) or CAL-101 (10 μM) alone and combined with SP600125 (20 μM) for 24 hr. Representative photographs showing the invasive cells that had passed through matrigel to the lower surface of the membrane at 100× magnification. (B-D) Invaded cells from 5 representative fields were counted. All of the drug combinations did not enhanced the inhibitory effect (n=2).


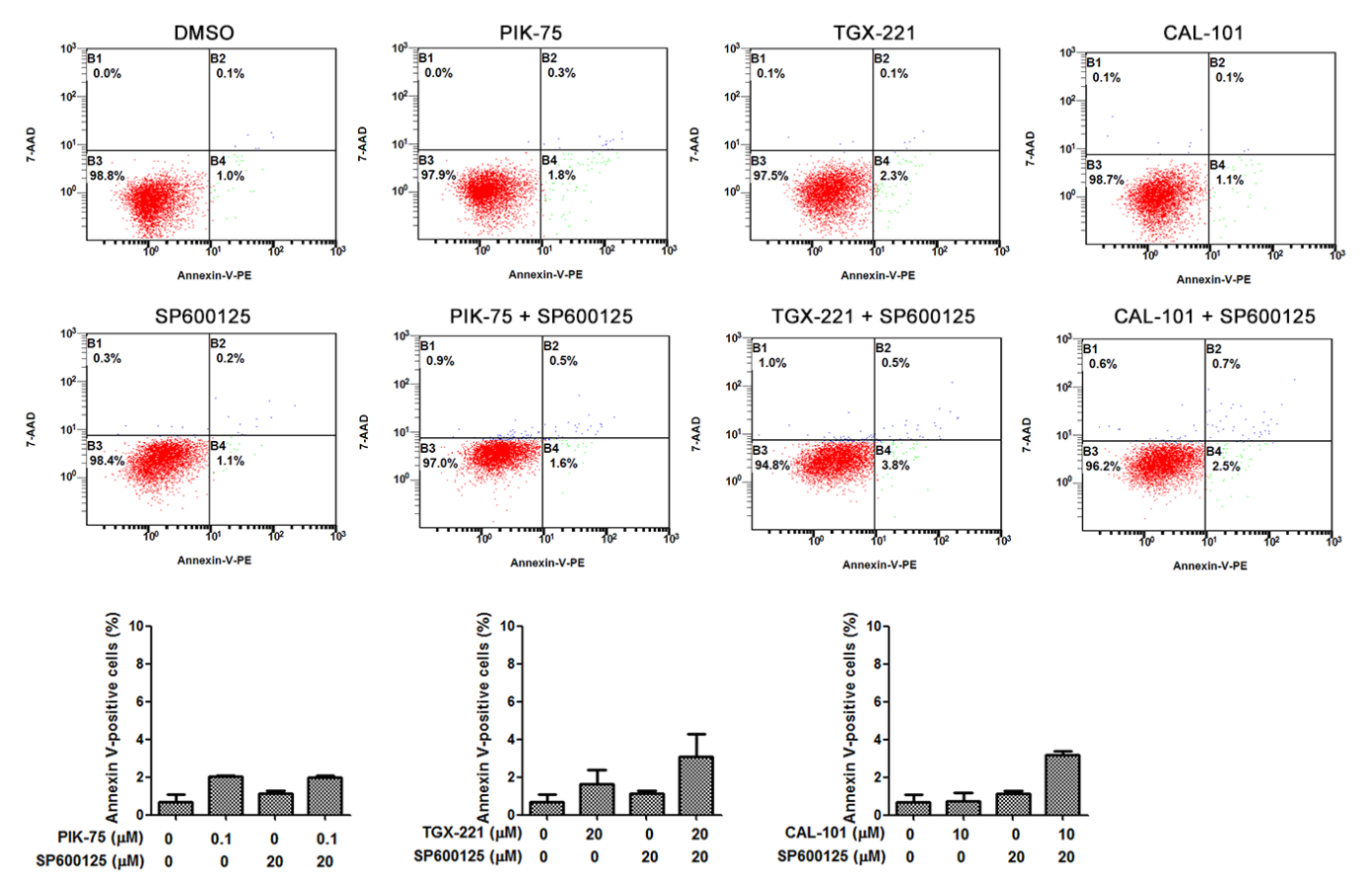


**Supplementary Figure 6: Combination effects of isoform-selective PI3K inhibitors and SP600125 on glioblastoma U-87 MG cell apoptosis.** U-87 MG cells were treated with PIK-75 (0.1 μM), TGX-221 (20 μM) or CAL-101 (10 μM) alone and combined with SP600125 (20 μM) for 48 hr. DMSO was used as a carrier control (n=2).
